# Low carbohydrate availability reduces power output at the moderate-to-heavy transition, impairs efficiency, and increases median power frequency during cycling in females

**DOI:** 10.1007/s00421-026-06260-3

**Published:** 2026-06-02

**Authors:** Evana Main, SangHoon Yoon, Samuel L. James, Kimberley M. Mellor, Matthew J. Brick, Warren B. Leigh, Ed Maunder

**Affiliations:** 1 School of Sport, Exercise, and Health, Faculty of Health and Environmental Sciences, Auckland University of Technology, Auckland, New Zealand; 2 Sports Performance Research Institute New Zealand, Auckland University of Technology, Auckland, New Zealand; 3 Department of Physiology, University of Auckland, Auckland, New Zealand; 4 Department of Anatomy and Physiology, University of Melbourne, Melbourne, VIC Australia; 5 Orthosports NZ, Auckland, New Zealand

**Keywords:** Carbohydrate availability, Intensity domain transitions, Cycling efficiency, Fibre recruitment, Female cyclists

## Abstract

**Purpose:**

Carbohydrate availability is reduced during prolonged exercise, and this may contribute to the loss of power output at the intensity domain transitions. The aim of this study was to investigate the effects of lowered carbohydrate availability on power output at intensity domain transitions, muscle activation, and gross cycling efficiency in endurance-trained female cyclists.

**Methods:**

Nine well-trained female cyclists completed a randomised, counterbalanced crossover study consisting of two conditions. Participants completed an incremental cycling test and three-minute all-out test, preceded by glycogen-depleting exercise ~ 24 h beforehand and subsequent carbohydrate ingestion of either ≥ 9 g kg^−1^ (HIGH) or ≤ 1 g kg^−1^ (LOW).

**Results:**

Power output at the first ventilatory threshold was reduced in LOW (133 ± 24 vs. 152 ± 28 W, ∆ − 19 ± 14 W, *P* = 0.011), with no between-trial difference in power output at the lactate threshold. Gross cycling efficiency during submaximal cycling was reduced in LOW (*P* = 0.003). Electromyographic median power frequency of the vastus lateralis (*P* = 0.025) and vastus medialis (*P* = 0.007) was higher in HIGH during submaximal cycling, with no between-trial differences in electromyographic amplitude. There was no between-trial difference in end-test power output during the three-minute all-out test.

**Conclusion:**

These data suggest lowered carbohydrate availability reduced power output at the moderate-to-heavy transition, possibly due to increased recruitment of higher-threshold motor units to compensate for glycogen-depleted fibres, impairing gross cycling efficiency. These data suggest that carbohydrate availability is likely important in ‘durability’ of the moderate-to-heavy intensity transition.

## Introduction

The moderate, heavy, and severe exercise intensity domains are characterised by attainment of a rapid, delayed, or absent steady state, respectively, in a range of metabolic variables such as phosphocreatine (PCr) and pH, blood lactate concentrations, and whole-body oxygen uptake (Burnley and Jones 2018; Jones et al. 2008, 2019; Poole et al. 2016; Vanhatalo et al. 2016; Chidnok et al. 2013). Power output at the intensity domain transitions is routinely assessed in a well-rested state and used to inform training programming and load monitoring (Maunder et al. 2021; Joyner and Coyle 2008). However, power output at the intensity domain transitions decreases non-linearly during prolonged exercise (Barrett and Maunder 2025; Clark et al. 2019a, b; Gallo et al. 2024; Hamilton et al. 2024; Stevenson et al. 2024), resilience to which is termed ‘durability’ (Maunder et al. 2021). Durability has implications for the application of physiological assessment data, as exercise in the moderate, heavy, and severe intensity domains elicits distinct metabolic, autonomic, and adaptive responses (Black et al. 2017; Seiler et al. 2007).

Durability of the intensity domain transitions could be related to carbohydrate availability. The ability to sustain prolonged work is linked to muscle glycogen (Nielsen et al. 2024; Alghannam et al. 2016). Muscle glycogen levels decrease progressively during exercise (Hermansen et al. 1967; Bergström et al. 1967), and muscle glycogen depletion is associated with fatigue (Bangsbo et al. 1992; Ørtenblad et al. 2011, 2013). Lowered muscle glycogen is linked to impaired Na⁺/K⁺-ATPase activity, disrupted Ca²⁺ handling, and reduced muscle contractile function (Ørtenblad et al. 2013; Duhamel et al. 2006a; Jensen et al. 2020a). Type I fibres are preferentially activated during prolonged exercise, and therefore are glycogen-depleted first (Nielsen et al. 2024). Thus, muscle glycogen depletion requires recruitment of higher-threshold motor units, or increased firing rate of active motor units, to maintain a given work rate (Tenan and Blackburn 2016). Importantly, type II fibres have poorer energetic efficiency than type I fibres (Coyle et al. 1992; Horowitz et al. 1994). Therefore, exercise-induced muscle glycogen depletion may contribute to decreased power output at intensity domain transitions via impairment of muscle contractile function, increased type II motor unit recruitment, and reduced energetic efficiency. Supporting a role for carbohydrate availability in durability, carbohydrate ingestion mitigates the loss of power output at the intensity domain transitions during prolonged cycling (Clark et al. 2019a, b; Dudley-Rode et al. 2025). However, the effect of glycogen availability on power output at the intensity domain transitions, and therefore exercise-induced muscle glycogen depletion in durability, has not been clearly established, having only been studied using associational designs (Clark et al. 2019b).

Accordingly, the primary aim of this study was to determine the effect of reduced carbohydrate availability on power output at the intensity domain transitions in female cyclists, and our secondary aims were to explore effects on muscle activation and gross efficiency during submaximal cycling. We hypothesised that lowered carbohydrate availability would reduce power output at intensity domain transitions, increase recruitment of type II fibres at submaximal power outputs, increasing muscle activation, and reduce submaximal gross efficiency. We investigated these questions in female cyclists due to the lack of research conducted in female populations (Ranadive and Hagberg 2025).

## Methods

### Participants

Nine trained female endurance cyclists and triathletes participated in the present investigation (${\dot{\mathrm{V}}}$O_2_peak, 44.3 ± 5.9 mL kg^−1^ min^−1^; mass, 62.4 ± 8.4 kg; age, 38 ± 14 years). A priori sample size calculation determined that five participants are required to detect an effect size of 1.7 with 80% statistical power and an alpha value of 0.05 (G*Power, Aichach, Germany). This effect size was based on the effect of prolonged exercise on power output at the moderate-to-heavy intensity transition (Stevenson et al. 2024). We assumed the effect of lowered glycogen would be smaller than that of prolonged exercise, given prolonged exercise elicits numerous physiological effects that could influence power output at the intensity domain transitions beyond lowered glycogen. A sample size of nine is sufficient to detect an effect size of 1.1 with 80% statistical power and an alpha value of 0.05. All participants were free of recent (< 3 months) illness and musculoskeletal injury and free of cardiovascular disease. Participants met at least two of three criteria: cycling training > 5 h.week^−1^, peak oxygen uptake (${\dot{\mathrm{V}}}$O_2_peak) > 48 mL kg^−1^ min^−1^, and self-reported best-effort 20-min power output of > 3.0 W kg^−1^. All procedures were approved by the Auckland University of Technology Ethics Committee (24/247), participants provided written informed consent and completed a general health screening. Menstrual cycle phase and oral contraceptive use were not controlled as these factors do not significantly influence physiological profiling variables in trained females (James et al. 2023; Mattu et al. 2020; Williams et al. 2023).

### Study design

**Fig. 1:**
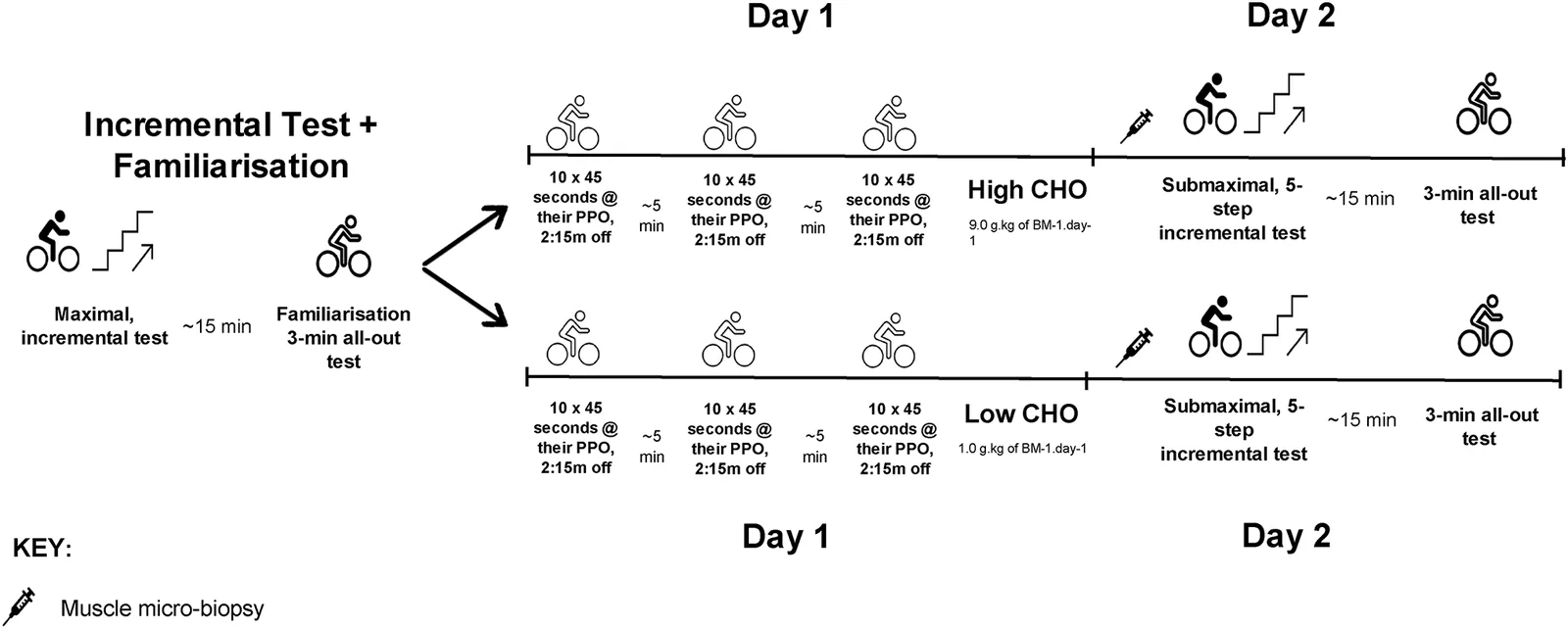
Schematic overview of the randomised crossover study design. Participants completed the “High CHO” and “Low CHO” crossover trials 5–14 days apart, in random order. High CHO, high carbohydrate diet; low CHO, low carbohydrate diet. PPO, peak power output achieved during the maximal, incremental test

An overview of the randomised, crossover study design is shown in Fig. 1. Participants visited the laboratory on five occasions: (1) a characterisation trial, which included an incremental cycling test to determine participant eligibility, and a familiarisation to the three-minute all-out test, (2) glycogen-depleting exercise, (3) an experimental trial including a comprehensive physiological profiling assessment of VT_1_, critical power, ${\dot{\mathrm{V}}}$O_2_peak, and cycling economy, (4) glycogen-depleting exercise, (5) the crossover experimental trial. Between glycogen-depleting exercise bouts and the experimental trials (~ 24 h), participants consumed a low (< 1 g kg BM^−1^) or high (> 9 g kg BM^−1^) carbohydrate diet to produce two distinct glycogen storage conditions (Tarnopolsky et al. 2001).

### Visit one: characterisation trial

#### Incremental test

Participants reported to the laboratory having fasted overnight for ~ 10 h and having refrained from alcohol and caffeine consumption for ~ 18 h. After providing written informed consent, height and body mass were determined. Cycling commenced with a 5-min warm-up at 50 W on an electromagnetically-braked cycle ergometer (Lode; Excalibur sport, Groningen, The Netherlands). Subsequently, the incremental cycling test began at 75 W, with power output increasing by 25 W every 3 min. Expired gases and heart rate were collected continuously using indirect calorimetry (TrueOne 2400, ParvoMedics, UT, USA) and a chest-strap heart rate monitor (Polar Electro Oy, Kempele, Finland). When clear signs of increased ${\dot{\mathrm{V}}}$_E_ ${\dot{\mathrm{V}}}$O_2_^−1^ emerged, power output was increased by 25 W every minute. The ${\dot{\mathrm{V}}}$O_2_peak was accepted as the highest 15-s average ${\dot{\mathrm{V}}}$O_2_, and VT_1_ was identified as the first breakpoint in the ${\dot{\mathrm{V}}}$O_2_ vs. ${\dot{\mathrm{V}}}$_E_. ${\dot{\mathrm{V}}}$O_2_ ^−1^ relationship. This ${\dot{\mathrm{V}}}$O_2_ was converted to power output by linear regression of the ${\dot{\mathrm{V}}}$O_2_ vs. power output relationship, using the last minute of ${\dot{\mathrm{V}}}$O_2_ data from each 3-min stage. The last minute of expired gas data in each 3-min stage was also used to quantify whole-body carbohydrate and fat oxidation rates using standard equations (Jeukendrup and Wallis 2005). The highest fat oxidation rate was identified as the peak fat oxidation rate (Maunder et al. 2022).

#### Three-minute all-out test familiarisation

After completing the incremental test, participants rested for ~ 15 min before completing a familiarisation to the 3-min all-out test. The 3-minute all-out test began with participants performing a warm-up at 100 W for 5 min. Participants then rested for 30 s and, during the last 5 s, increased cadence to approximately 120 rev.min^−1^. Participants then cycled all-out for 3 min. Participants were instructed to reach peak power output as quickly as possible and maintain an all-out effort throughout. Loud verbal encouragement was given, but the subjects were not informed of the elapsed time. The resistance to pedalling during the test was set so that participants attained a power output of 50% of the difference between VT_1_ and ${\dot{\mathrm{V}}}$O_2_peak at 67.5 rev.min^−1^, using the linear factor (linear factor = power/cadence^2^) (Burnley et al. 2006; Vanhatalo et al. 2007). Expired gases and heart rate were collected throughout. End-test power (analogous to critical power) was calculated as the mean power output in the last 30-s of the 3-min all-out test. End-test power output is a valid measure of the heavy-to-severe intensity transition (Vanhatalo et al. 2007). Following the 3-min all-out test, a consultation occurred in which preferred foods were discussed to create a draft meal plan. Participants were made familiar with the smartphone application used throughout the study to track macronutrient intake (Easy Diet Diary).

### Visits two and four: glycogen-depleting exercise

Participants returned ~ 5–14 days later to complete glycogen-depleting exercise. Participants arrived having fasted overnight for ~ 10 h and refrained from alcohol and caffeine consumption for ~ 18 h. Cycling began with a 5-min warm-up at 75 W. Participants then completed 10 × 45-s intervals at the highest power achieved during the incremental test in the characterisation trial, with 135-s of passive recovery between repetitions. Participants then rested passively for 5-min. This sequence was repeated two further times for a total of 30 repetitions (Vigh-Larsen et al. 2022).

Following the glycogen-depleting exercise, < 1.0 g kg BM^−1^ (LOW) or > 9 g kg BM^−1^ (HIGH) carbohydrate was consumed until the subsequent experimental session (~ 24 h) (Tarnopolsky et al. 2001). The order of LOW and HIGH was randomised. In both trials, target energy intake was 100–133% of the calories provided by 9 g kg BM^−1^ of carbohydrate.

### Visits three and five: experimental sessions

#### Muscle microbiopsy

On the morning of the experimental session, participants returned to the laboratory, having consumed a light breakfast containing of 0.2 g kg of BM^− 1^ carbohydrates. This breakfast was replicated in both trials. A resting microbiopsy muscle sample was obtained from the mid-belly of the vastus lateralis of the dominant leg. Local anaesthesia was applied to the skin and superficial muscle fascia. A microbiopsy needle was then inserted ~ 2 cm into the mid-belly of the vastus lateralis to recover ~ 15–30 mg of tissue using a spring-loaded mechanism (14G Ultimate, Zamar, Croatia). Muscle tissue was immediately frozen on dry ice and stored at − 80 °C until further analysis.

#### Measurement of muscle activity

Muscle activity was measured via surface electromyography (Quattrocento, OT Bioelettronica S.r.l., Torino, Italy, sampled at 2048 Hz and bandpass filtered 20–500 Hz). Before the start of the test, location of the motor point of the VL and VM muscles was estimated using a stimulation pen, which was used to deliver single rectangular pulse stimuli (duration = 0.2 ms, intensity = 20 mA) by a high-voltage constant current stimulator (DS7AH; Digitimer, Welwyn Garden City, UK). The motor point was that which provoked the strongest twitch, as demonstrated by visual inspection by the researcher and the contraction sensation of the participants. A self-adhesive 5 × 10 cm rectangle electrode was placed on the gluteal fold. The disposable electromyography (EMG) electrodes (Norotrode, Myotronics Inc., WA, USA; 22 mm inter-electrode distance) were placed such that the proximal electrode was over the motor point and the electrodes were oriented in the direction of the muscle fibres. The ground electrode was placed on the bony region of the shin. Before placing the electrodes, the skin was shaved and abraded with alcohol. The EMG electrode placement locations were outlined with a marker for replication during the second experimental trial.

#### Experimental trial

The exercise trial began with a five-step incremental test, where the first step was at a power output 20% below the VT_1_ power output estimated in the characterisation trial. The power output was then increased by 10% of the previously estimated VT_1_ every 4 min, such that the fifth and final step was 20% above the VT_1_ power output estimated in the first visit. A finger capillary blood sample was obtained in the last 30 s of each stage for lactate analysis via a hand-held lactate analyser (Lactate Pro, Akray, Japan). Heart rate and expired gases were collected continuously. Raw EMG signals were recorded for the VL and VM. Participants then rested for 15 min before completing the three-minute all-out test according to the procedures described in the characterisation trial. Loud verbal encouragement was given throughout. These experimental procedures were repeated for the other condition ~ 7 days later (9 ± 3).

### Data analysis

#### Five-step test

The VT_1_ power output was calculated using similar methods to those used during the first laboratory visit. Specifically, ${\dot{\mathrm{V}}}$O_2_ at VT_1_ was identified as the first breakpoint in the ${\dot{\mathrm{V}}}$O_2_ vs. ${\dot{\mathrm{V}}}$_E_. ${\dot{\mathrm{V}}}$O_2_ ^−1^ relationship. This ${\dot{\mathrm{V}}}$O_2_ was converted to power output by linear regression of the ${\dot{\mathrm{V}}}$O_2_ vs. power output relationship, using the last minute of ${\dot{\mathrm{V}}}$O_2_ data from each 4-min stage. This method has previously produced similar results to blood lactate-derived measurements (Stevenson et al. 2022). The final sample size for this measure was *N* = 7, as clear evidence of VT_1_ was not observed for two participants. We repeated these analyses for the gas exchange threshold using the V-slope method, whereby the ${\dot{\mathrm{V}}}$O_2_ at the first breakpoint in the ${\dot{\mathrm{V}}}$O_2_ vs. ${\dot{\mathrm{V}}}$CO_2_ relationship (Jamnick et al. 2020). To estimate lactate threshold, blood lactate concentrations during the five-step test were plotted against power output, and the breakpoint in the curve was identified using the LoglogLT method (Jamnick et al. 2018). Expired gas data were used to quantify rates of whole-body energy expenditure, carbohydrate oxidation, and fat oxidation using the last minute of expired gas in each 4-min stage and standard equations (Jeukendrup and Wallis 2005). Gross efficiency was calculated as the percentage of metabolic energy expenditure converted to mechanical power using the energy expenditure calculated in the last minute of each stage.

The mechanical power output achieved at an intensity domain transition is, mathematically, determined by the rate of metabolic energy expenditure achieved at the transition (here termed ‘metabolic power’) and the efficiency of conversion of that metabolic energy expenditure to mechanical power output (here termed ‘metabolic efficiency’). We therefore quantified the proportion of between-trial differences in power output at the intensity transitions attributable to changes in metabolic efficiency and metabolic power. We first estimated metabolic power at VT_1_, lactate threshold, and end-test power output in LOW by linear regression of the mechanical power output (W) vs. metabolic energy expenditure (kcal min^−1^) relationship in LOW. We then used metabolic power at VT_1_, lactate threshold, and end-test power output values in LOW to estimate the power output at the transitions that would be achieved with the metabolic efficiency in HIGH (here termed ‘LOW_Power_HIGH_Eff_’). To estimate LOW_Power_HIGH_Eff_, we converted metabolic power in LOW (kcal min^−1^) to mechanical power output using linear regression of the mechanical power output (W) vs. metabolic energy expenditure (kcal min^−1^) relationship in HIGH. Accordingly, the proportion of LOW-induced changes in power output at the intensity domain transitions attributable to changes in metabolic efficiency and metabolic power was calculated using the below equations (Eq. 1).

1 $$\begin{aligned} & {\mathrm{Contribution}}\;{\mathrm{of}}\;{\mathrm{metabolic}}\;{\mathrm{efficiency}}\;{\mathrm{to}}\;{\mathrm{between-trial}}\;{\mathrm{difference}} \\ & \quad {\text{in power}}\;{\mathrm{output}}\;{\mathrm{at}}\;{\mathrm{the}}\;{\mathrm{intensity}}\;{\mathrm{domain}}\;{\mathrm{transition}} \\ & \quad = {\text{LOW }}-{\mathrm{LOW}}_{{{\mathrm{Power}}}} {\mathrm{HIGH}}_{{{\mathrm{Eff}}}} \\ & {\mathrm{Contribution}}\;{\mathrm{of}}\;{\mathrm{metabolic}}\;{\mathrm{power}}\;{\mathrm{to}}\;{\mathrm{between-trial}}\;{\mathrm{difference}} \\ & \quad {\text{in power}}\;{\mathrm{output}}\;{\mathrm{at}}\;{\mathrm{the}}\;{\mathrm{intensity}}\;{\mathrm{domain}}\;{\mathrm{transition}} \\ & \quad = {\mathrm{LOW}}_{{{\mathrm{Power}}}} {\mathrm{HIGH}}_{{{\mathrm{Eff}}}} - {\mathrm{HIGH}}{\mathrm{.}} \\ \end{aligned}$$

where LOW = power output at an intensity domain transition in LOW, HIGH = power output at an intensity domain transition in HIGH, and LOW_Power_HIGH_Eff_ = power output that would be produced in HIGH using the metabolic power at an intensity domain transition in LOW.

#### Three-minute all-out test

End-test power output was estimated as mean power output in the last 30-s of the 3-min all-out test. The work completed during the test above end power (WEP) was also calculated (Jones et al. 2010). Peak power was the highest 1-s value. The ${\dot{\mathrm{V}}}$O_2_peak was accepted as the highest 15-s average ${\dot{\mathrm{V}}}$O_2_.

#### Muscle activation

Custom programs in MATLAB (The MathWorks, Inc., Natick, MA, USA) were used to analyse EMG data. EMG data were band-pass filtered (Butterworth 4th order, 20–450 Hz) and partitioned into five bins for the five-step test (i.e., five stages), and into six bins for the three-min all-out test (i.e., 30-s bins). For the five-step test, only data from the last-minute each stage were analysed. EMG data were available for seven participants due to data loss from a system disconnection and a detached ground electrode.

The filtered data were full-wave rectified, and root mean squared (RMS) with a 25-ms moving window. Based on prior research (Özgünen et al. 2010), we used 35% of the mean value calculated over the whole RMS envelope during the five-step test and three-min all-out test to identify active bursts in the VL and VM during the respective tests (Yoon et al. 2025). The EMG data under the burst threshold were excluded from the analyses. Median power frequency (MPF) was calculated as the frequency that divides the power spectral density (PSD) into two equal halves, using the periodogram-based method.

To normalise RMS and MPF data, peak values were obtained from the three-min all-out test. The three-min all-out test data were partitioned into 1-s bins and the highest RMS and MPF values were used for normalisation. Stage-specific RMS and MPF values from the five-step test and 30-s bin values from the three-min all-out test were expressed relative to these peak values.

### Muscle glycogen analysis

Frozen muscle tissue samples were homogenised in a buffer containing 100 mM Tris–HCl, 5 mM EGTA, and 5 mM EDTA (Sigma Aldrich, MO, USA), supplemented with Halt protease and phosphatase inhibitor cocktail (ThermoFisher Scientific, Waltham, MA). The protein concentration of the homogenates was determined in triplicate using a Lowry assay. Glycogen concentration was measured by digesting an aliquot of the crude homogenate with amyloglucosidase (Sigma-Aldrich, St. Louis, MO, USA) at 50 °C for 60 min in a buffer containing 0.1 M sodium acetate, pH 6.0. An additional aliquot of crude homogenate was processed in parallel without amyloglucosidase to account for background free glucose. Following centrifugation at 16,000 g for 2 min, the supernatant was removed, and glucose levels were determined in triplicate using a two-enzyme, colorimetric glucose assay (Sigma-Aldrich, St. Louis, MO, USA). Glycogen levels were measured in glycosyl units and normalised to protein content and dry weight.

### Statistical analysis

Data are expressed as means ± standard deviation. The Shapiro–Wilk test was used to confirm a normal Gaussian distribution. Power output at VT_1_ and lactate threshold, end-test power, muscle glycogen content, ${\dot{\mathrm{V}}}$O_2_peak, WEP, peak power, and total work done during the 3-min all-out test were compared between-trials using paired t tests. Linear mixed models were used to assess substrate oxidation and EMG data. Condition, stage and their interaction were included as fixed effects, and participant ID was included as a random effect to account for within-subject variability. Where a significant interaction effect was observed, we used Holm-Bonferroni post-hoc tests to locate variance. Mean absolute differences (± standard deviations) are presented where appropriate. Cohen’s d_z_ effect sizes (± 95% confidence intervals) are presented where appropriate. The α for all statistical tests was set at *P* < 0.05. Analyses were conducted using JASP (version 0.19.3).

## Results

### Diet and muscle glycogen content

Participants consumed significantly more carbohydrate (470 ± 141 vs. 74 ± 21 g, *P* ≤ 0.001) and total energy (3249 ± 636 vs. 2606 ± 539 kcal, *P =* 0.002), with less protein (122 ± 37 vs. 200 ± 46 g, *P* = 0.003) and fat (98 ± 43 vs. 168 ± 48 g, *P* = 0.003), between glycogen-depleting exercise and the experimental trial in HIGH than in LOW. Muscle glycogen was significantly lower in LOW than HIGH when normalised to protein (447 ± 157 vs. 731 ± 174 nmol glycosyl units mg^−1^ protein, *P* = 0.005) and dry weight (248 ± 97 vs. 406 ± 110 nmol glycosyl units mg^−1^ dw, *P* = 0.005).

### Intensity domain transitions

**Fig. 2:**
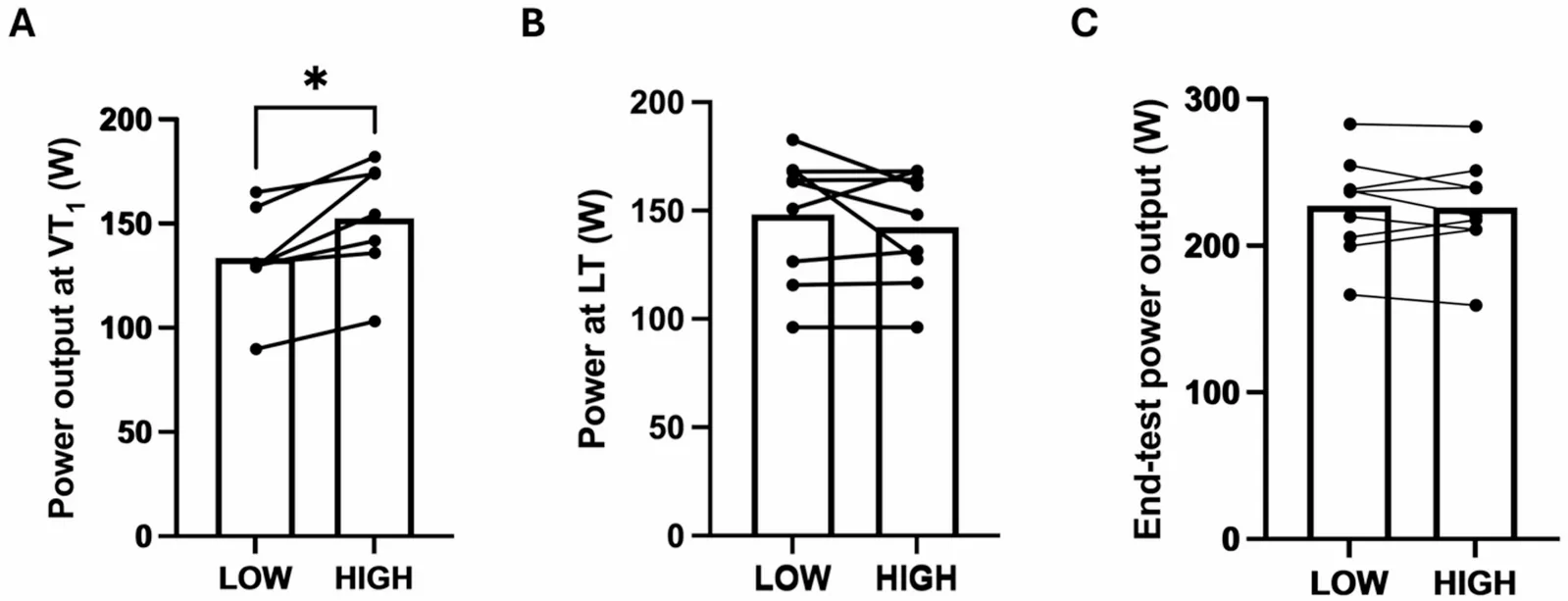
Power output at the moderate-to-heavy intensity transition as determined by the **A** first ventilatory threshold (VT_1_) (*N* = 7) and **B** lactate threshold (LT) (*N* = 9) in a low carbohydrate (LOW) and high (HIGH) carbohydrate state. **C** End test power (EP) during the three-minute all-out test in a low (LOW) and high (HIGH) carbohydrate state (*N* = 9). Data were analysed using paired t tests. Bars indicate mean values and lines indicate individual responses. *P = 0.011

Power output at VT_1_ was significantly lower in LOW than HIGH (133 ± 24 vs. 152 ± 28 W, ∆ − 19 ± 14 W, d_z_ = − 1.37 [− 2.71, − 0.03], *P* = 0.011, Fig. 2A, *N* = 7). The rate of energy expenditure at VT_1_ was not significantly different between conditions (9.1 ± 1.5 vs. 9.8 ± 1.5 kcal min^−1^ in LOW and HIGH respectively, ∆ 0.7 ± 0.8, *P* = 0.067). The change in power output at VT_1_ from HIGH to LOW was attributable to decreased metabolic efficiency (− 7 ± 8 W) and metabolic power (− 12 ± 15 W). The contribution made by decreased metabolic efficiency and metabolic power to the decrease in power output at VT_1_ was not significantly different (*P* = 0.525). Power output at the gas exchange threshold was lower than VT_1_, but also significantly lower in LOW than HIGH (110 ± 22 vs. 140 ± 26 W, respectively, *P* < 0.0001). For ease of interpretation, from here on we refer only to VT_1_. Power output at the lactate threshold was not significantly different between-conditions (148 ± 29 vs. 142 ± 26 W, ∆ 6 ± 19 W, d_z_ = 0.08 [− 0.69, 0.85], *P* = 0.331, Fig. 2B, *N* = 9).

End-test power was not significantly different between-conditions (227 ± 34 vs. 226 ± 34 W in LOW and HIGH, respectively, ∆ 1 ± 12 W, d_z_ = 0.11 [− 0.66, 0.88], *P* = 0.748, Fig. 2C, *N* = 9). Similarly, there was no significant effect of condition on WEP (8.6 ± 2.2 vs. 9.3 ± 2.3 kJ in LOW and HIGH respectively, *P* = 0.232), ${\dot{\mathrm{V}}}$O_2_peak (2.8 ± 0.3 vs. 2.8 ± 0.4 L.min^−1^ in LOW and HIGH respectively, *P* = 0.434, *N* = 9), peak power output (846 ± 194 W vs. 864 ± 169 W in LOW and HIGH respectively, *P* = 0.554, *N* = 9), or total work done during the three-minute all-out test (49.7 ± 6.97 kJ vs. 50.2 ± 6.94 kJ in LOW and HIGH respectively, *P* = 0.192, *N* = 9).

### Muscle activity

**Fig. 3:**
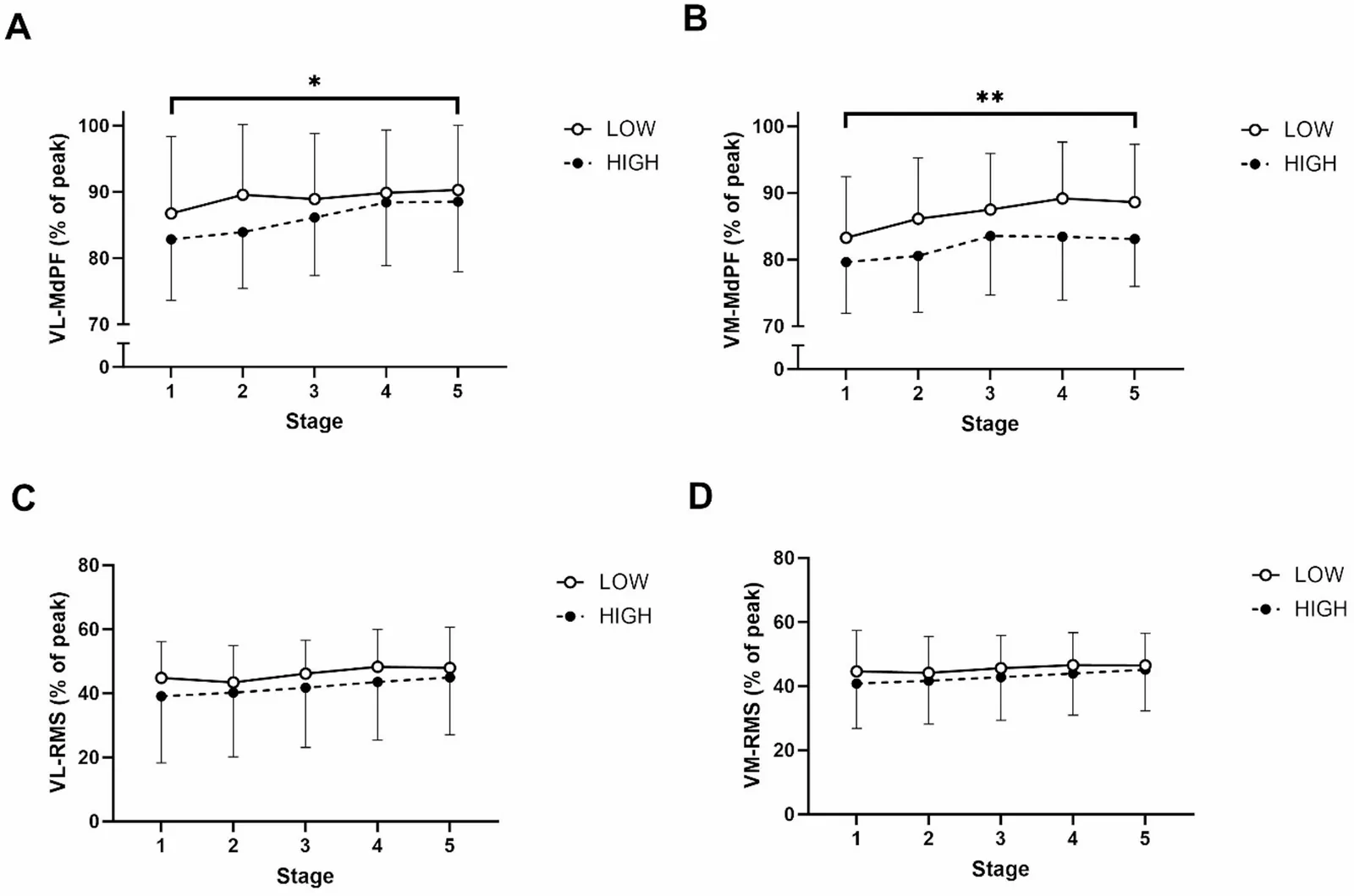
Muscle activity during the five-step test was determined by **A** Vastus lateralis median power frequency (MPF), **B** vastus medialis median power frequency (MPF), **C** vastus lateralis amplitude (RMS), **D** vastus medialis amplitude (RMS) during the five-step test. Data are normalised to the peak value achieved during the corresponding three-minute all-out test and analysed using linear mixed models. The dots indicate raw means, and the error bars indicate SD. * denotes a condition effect *P* < 0.05. ** denotes a condition effect *P* < 0.01. *N* = 7

During the five-step test, median power frequency of the VL (d_z_ = 0.48 [− 0.51, 1.46], *P* = 0.025) and VM (d_z_ = 0.95 [− 0.14, 2.23], *P* = 0.007) was significantly higher in LOW than HIGH, with no condition-by-stage interaction for VL (*P* = 0.375) or VM (*P* = 0.749) (Fig. 3A, B, *N* = 7). There was no effect on amplitude in the VL (*P* = 0.232) and VM (*P* = 0.655), and there were no condition-by-stage interactions for VL (*P* = 0.809) or VM (*P* = 0.830, Fig. 3C, D, *N* = 7). There was no significant effect of condition for amplitude or median power frequency in the VL and VM during the 3-min all-out test (data not shown).

### Substrate oxidation

**Fig. 4:**
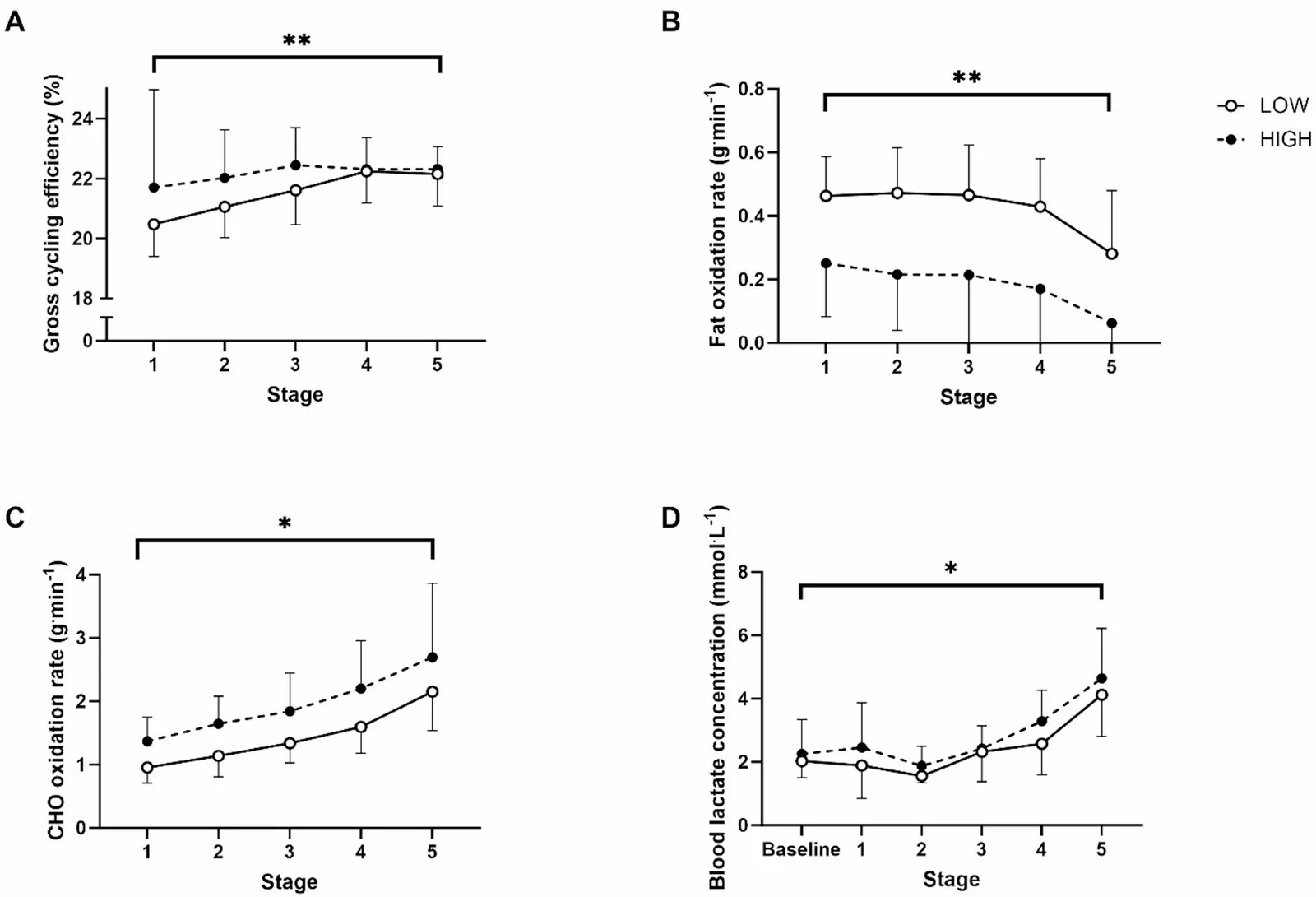
A Gross efficiency, **B** fat oxidation rates, **C** carbohydrate oxidation rates, and **D** blood lactate concentrations during the five-step test. Data were analysed using linear mixed models. The dots indicate raw means, and the error bars indicate SD. * denotes a condition effect *P* < 0.05. ** denotes a condition effect *P* < 0.01. *N* = 9

Gross efficiency during the five-step test was significantly lower in LOW than HIGH (d_z_ = − 0.57 [− 1.40, 0.27], *P* = 0.003), and there was a condition-by-stage interaction (*P* = 0.041), although there were significant pairwise comparisons (Fig. 4A, *N* = 9). Fat oxidation rates during the five-step test were significantly higher in LOW than HIGH (*P* = 0.006), but there was no condition-by-stage interaction (*P* = 0.799, Fig. 4B). Carbohydrate oxidation rates during the five-step test were significantly lower in HIGH than LOW (*P* = 0.016), but there was no condition-by-stage interaction (*P* = 0.734, Fig. 4C). Blood lactate concentrations during the five-step test were significantly lower in LOW than HIGH (*P* = 0.023), but there was no condition-by-stage interaction (*P* = 0.880, Fig. 4D).

## Discussion

Our acute exercise-diet manipulation protocol successfully produced two distinct muscle glycogen storage conditions. We observed that lowered carbohydrate and muscle glycogen availability in LOW: (i) reduced power output at VT_1_, but not the lactate threshold or critical power, (ii) increased electromyographic median power frequency, but not amplitude, of the VL and VM during submaximal cycling, and (iii) reduced gross efficiency during submaximal cycling. These data suggest reduced carbohydrate substrate availability may reduce power output at the moderate-to-heavy, but not heavy-to-severe intensity transition, due to alterations in motor unit recruitment and impaired gross cycling efficiency. Specifically, our data are consistent with additional recruitment of high-threshold motor units at a given submaximal power output under conditions of lowered carbohydrate availability, and support a mechanistic role for the progressive reduction of muscle glycogen content during prolonged exercise in durability of the moderate-to-heavy intensity transition.

### Loss of power output at the first ventilatory threshold with reduced carbohydrate availability

Consistent with our hypothesis, lowered carbohydrate availability reduced power output at VT_1_ by ~ 12% (Fig. 2A) and impaired gross efficiency (Fig. 4A) in LOW. This occurred alongside an ~ 39% lower whole-muscle glycogen concentration and evidence of greater recruitment high-threshold motor units. Specifically, we observed significantly higher VL and VM median power frequency in LOW (Fig. 3A, B). Activation of higher-threshold motor units, which have faster muscle fibre conduction velocities (Luca 1984), results in a shift in the electromyographic power spectrum toward higher frequencies, which increases the median power frequency (Vecchio et al. 2018; Masuda and Luca 1991). Therefore, the elevated VL and VM median power frequency at submaximal power outputs in LOW likely reflects increased activation of higher-threshold motor units. This is consistent with the reduction in gross cycling efficiency in LOW, as high-threshold, type II fibres are less energetically-efficient than low-threshold, type I fibres (Coyle et al. 1992; Horowitz et al. 1994).

Plausibly, the increased activation of higher-threshold motor units in LOW may have been caused by preferential glycogen depletion in low-threshold motor units during our glycogen-depleting exercise, with minimal repletion of these fibres during the subsequent ~ 24-h period of low carbohydrate intake in LOW (< 1 g kg^−1^). Previous studies of prolonged exercise have reported preferential glycogen depletion in type I fibres (Nielsen et al. 2024). Reduced muscle glycogen content impairs muscle contractile function via reduced sarcoplasmic reticulum Ca^2+^ release (Ørtenblad et al. 2013; Ørtenblad et al. 2011; Duhamel et al. 2006a, b) secondary to impaired function of Na^+^/K^+^-ATPase and ryanodine receptors (Jensen et al. 2020b; Chin and Allen 1997). Accordingly, lowered glycogen content and impaired contractile activity in low-threshold motor units in LOW may have necessitated a compensatory increase in recruitment of high-threshold motor units to produce a specific submaximal power output. However, we cannot confirm this hypothesis in the absence of fibre-specific muscle glycogen data, and recommend that future studies are conducted to compare power output at the moderate-to-heavy intensity transition and muscle activation under conditions of experimentally-manipulated muscle glycogen content, with measurement of muscle glycogen content in type I and type II fibres.

Interestingly, there was no significant effect of reduced carbohydrate availability on VL and VM amplitude (Fig. 3C, D). Our amplitude measure during submaximal cycling is related to the number of active motor units and firing rate (Goswami et al. 2025, Viitasalo and Komi 1977), although it should be interpreted with caveats (Farina et al. 2010). Therefore, we expected that increased recruitment of high-threshold motor units in LOW would increase VL and VM amplitude. It is possible that an increase in VL and VM amplitude associated with increased recruitment of high-threshold motor units in LOW was counterbalanced by faster conduction velocity related to reduced glycolytic flux. Whole-body carbohydrate oxidation rates and absolute blood lactate concentrations were lower during the submaximal incremental test in LOW (Fig. 4C, D). This is consistent with prior research reporting that reduced muscle glycogen content downregulates muscle glycogenolysis (Hargreaves et al. 1995). Reduced glycogenolysis mitigates the accumulation of intramuscular H^+^ (Thomassen et al. 2025). Lower intramuscular H^+^ accumulation is associated with faster conduction velocity (Brody et al. 1991). This can reduce motor unit action potential duration and area, resulting in less overlap and therefore reduced electromyographic amplitude (Keenan et al. 2005). Thus, reduced rates of muscle glycogenolysis in LOW may have placed downward pressure on VL and VM amplitude that counterbalanced the increase associated with increased recruitment of high-threshold motor units. This could explain the absence of a statistical difference in VL and VM amplitude between LOW and HIGH (Fig. 3C-D). However, it is also possible that we did not have sufficient statistical power to detect a small or moderate magnitude difference in VL and VM amplitude, as our study was powered for a large effect. Accordingly, our VL and VM amplitude data should be interpreted with caution.

Our data should be interpreted cautiously in light of the modest statistical power and unintentional discrepancy in total energy intake between the glycogen-depleting bout and the experimental trial in HIGH and LOW, whereby participants consumed ~ 25% more energy in HIGH. If participants were in negative energy balance in LOW but not HIGH, it is possible that parameters such as VT_1_ were affected independent of carbohydrate availability. However, energy intake in LOW averaged 40.9 ± 8.3 kcal kg⁻¹ day⁻¹, which is within the range commonly reported for endurance athletes and above thresholds typically associated with clinically meaningful low energy availability. Experimental and consensus data indicate that physiological and performance impairments are most consistently observed when energy availability falls below ~ 30 kcal kg fat-free mass⁻¹ day⁻¹ (Loucks and Thuma 2003; Mountjoy et al. 2018). Whilst we did not measure fat-free mass, energy intake in LOW must be substantially higher than this threshold, given our value of 40.9 ± 8.3 kcal kg⁻¹ day⁻¹ is reported relative to whole-body mass. While we acknowledge that the ~ 25% difference in energy intake between conditions is a limitation, the high absolute energy intake in LOW makes it unlikely that insufficient energy availability alone explains the observed differences in power output at VT_1_.

### Disparity in the effects of lowered carbohydrate availability on the first ventilatory threshold and lactate threshold

Interestingly, reduced carbohydrate availability lowered power output at VT_1_, but not the lactate threshold (Fig. 2B). The lactate threshold and VT_1_ are used as markers of the moderate-to-heavy intensity transition (Jamnick et al. 2020), hence we anticipated these markers would respond similarly to reduced carbohydrate availability. This disparity may be due to sensitivity to different physiological signals. The VT_1_ is assessed via the ventilatory response to exercise, which is sensitive to input from mechanosensitive group III and metabosensitive group IV muscle afferents (Amann et al. 2010). Therefore, increased muscle contractile activity and/or greater disturbance to broad metabolic homeostasis at given submaximal workloads could trigger a disproportionate increase in ${\dot{\mathrm{V}}}$_E_ relative to ${\dot{\mathrm{V}}}$O_2_ at a lower work rate, and therefore reduced power output at VT_1_ (Lam et al. 2019). In contrast, the lactate threshold is assessed solely via changes in blood lactate concentrations, and thus imbalances between lactate production and clearance (Faude et al. 2009; Goodwin et al. 2007). In LOW, we observed lower carbohydrate oxidation rates and absolute blood lactate concentrations (Fig. 4C, D), likely due to downregulated muscle glycogenolysis (Hargreaves et al. 1995), resulting in an unaltered power output vs. blood lactate relationship and therefore lactate threshold. However, increased high-threshold motor unit recruitment in LOW, evidenced by increased VL and VM median power frequency (Fig. 3A, B), may have resulted in greater stimulation of mechanosensitive group III muscle afferents, triggering an earlier disproportionate increase in ${\dot{\mathrm{V}}}$_E_ relative to ${\dot{\mathrm{V}}}$O_2_, and therefore the lower VT_1_. This could explain the reduction in VT_1_, with unchanged lactate threshold, in LOW.

### No effect of lowered carbohydrate availability on the heavy-to-severe intensity transition

Contrary to our hypothesis, reduced carbohydrate availability did not impact power output at the heavy-to-severe transition (Fig. 2C). This suggests factors other than carbohydrate availability govern the heavy-to-severe intensity transition, at least within the range of carbohydrate availabilities studied. In support, a previous study reported no relationship between the magnitude of muscle glycogen depletion during prolonged, heavy-intensity cycling and the magnitude of the reduction in power output at the heavy-to-severe intensity transition (Clark et al. 2019a). We made an error in our calculation of the linear factor fused for the three-minute all-out test, whereby the preferred cadence was fixed at 67.5 rev.min^−1^. However, we are confident that this did not impact our results, as three-minute all-out test end-test power output is robust to reductions in cadence below preferred values (Vanhatalo et al. 2008). A negative effect of reduced carbohydrate availability on power output at the heavy-to-severe intensity transition might be seen with greater reductions in muscle glycogen levels. It has been suggested that negative effects of muscle glycogen depletion on high-intensity performance are only seen when muscle glycogen falls below a threshold, possibly 250–300 mmol kg⁻¹ dw (Vigh-Larsen et al. 2021). Future studies should consider investigating the effects of more-severe muscle glycogen depletion on power output at the heavy-to-severe intensity transition.

Relatedly, a limitation of our study is the absence of resting muscle glycogen data obtained prior to the experimental exercise-diet protocol. Such data would allow us to directly ascertain if muscle glycogen concentrations were elevated or reduced compared to typical resting values in HIGH and LOW, and the magnitude of these effects. These data would aid interpretation. While there is a relative lack of data concerning resting muscle glycogen concentrations in female populations, our values in HIGH (406 ± 110 nmol glycosyl units mg^−1^ dw) are broadly similar to previous studies (385–545 nmol glycosyl units mg^−1^ dw), and, accordingly, our values in LOW (248 ± 97 nmol glycosyl units mg^−1^ dw) are lower (~ 35–55%) (Dawson et al. 2003; Devries et al. 2006; Hackney 1999). Therefore, these data support that our protocol successfully manipulated resting muscle glycogen concentrations to produce two distinct storage conditions, one of which being substantially lower than typical resting values.

## Conclusion

In conclusion, lowered carbohydrate availability reduced power output at the moderate-to-heavy transition, as indicated by reduced power output at VT_1_. We attribute this effect to greater activation of higher-threshold motor units during submaximal cycling, likely in compensation for impaired function of glycogen-depleted low-threshold motor units, resulting in impaired gross efficiency. No effect of reduced carbohydrate availability was observed at the heavy-to-severe transition. Our novel data therefore provide new insights into how lowered carbohydrate and muscle glycogen availability impact power output at the intensity domain transitions, and support a mechanistic role for the progressive reduction of carbohydrate availability during prolonged exercise in durability of the moderate-to-heavy intensity transition.

## Data Availability

Data is available from the corresponding author upon reasonable request.

## Abbreviations

${\dot{\mathrm{V}}}$O_2_peak: Peak rate of oxygen consumption
VL: Vastus lateralis
VM: Vastus medialis
VT_1_: First ventilatory threshold

## Code availability

None used.
