# Correction: Low carbohydrate availability reduces power output at the moderate-to-heavy transition, impairs efficiency, and increases median power frequency during cycling in females

**DOI:** 10.1007/s00421-026-06340-4

**Published:** 2026-07-13

**Authors:** Evana Main, SangHoon Yoon, Samuel L. James, Kimberley M. Mellor, Matthew J. Brick, Warren B. Leigh, Ed Maunder

**Affiliations:** 1 School of Sport, Exercise, and Health, Faculty of Health and Environmental Sciences, Auckland University of Technology, Auckland, New Zealand; 2 Sports Performance Research Institute New Zealand, Auckland University of Technology, Auckland, New Zealand; 3 Department of Physiology, University of Auckland, Auckland, New Zealand; 4 Department of Anatomy and Physiology, University of Melbourne, Melbourne, VIC Australia; 5 Orthosports NZ, Auckland, New Zealand

**Correction: European Journal of Applied Physiology (2026)** https://doi.org/10.1007/s00421-026-06260-3

In the original version of this article, Figure 2 was included twice as Figure 1 and Figure 2.

The Figure 1 should have appeared as shown below.

**Fig. 1:**
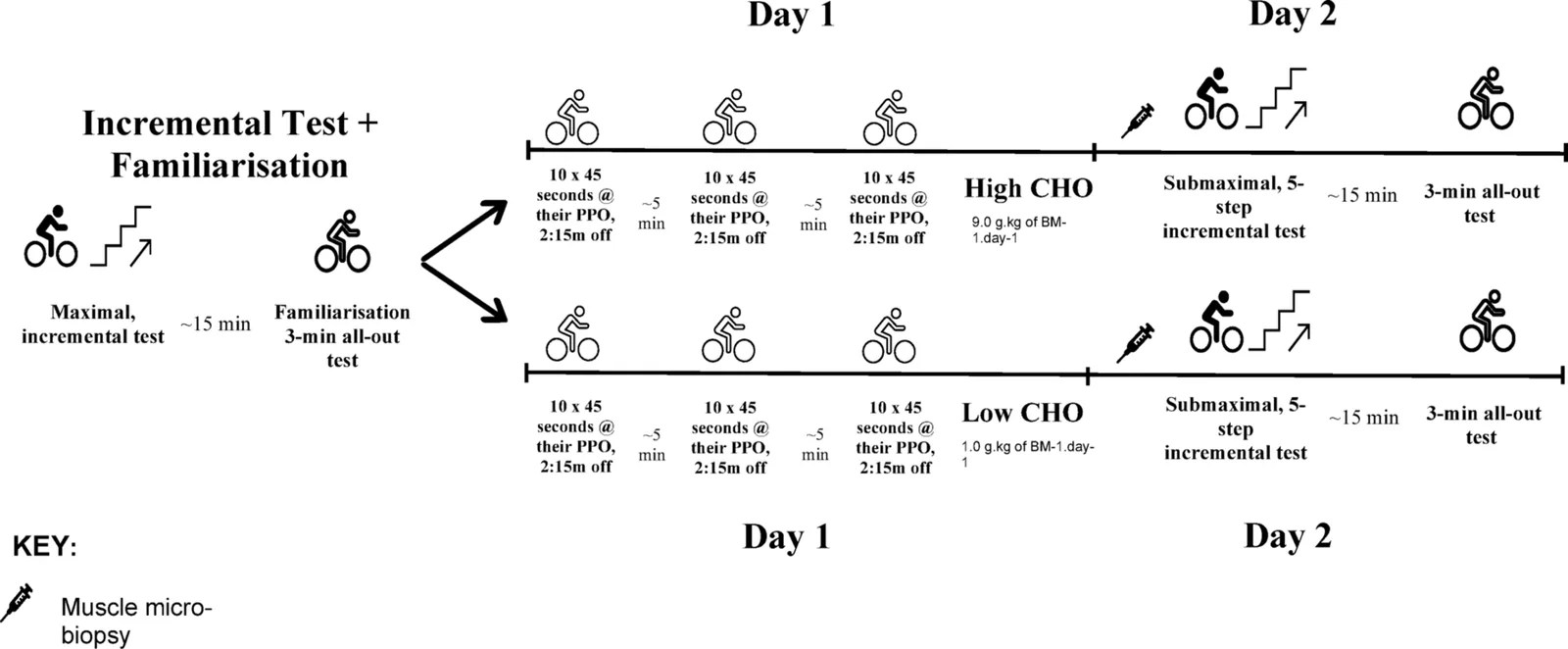
Schematic overview of the randomised crossover study design. Participants completed the “High CHO” and “Low CHO” crossover trials 5-14 days apart, in random order. High CHO, high carbohydrate diet; low CHO, low carbohydrate diet. PPO, peak power output achieved during the maximal, incremental test

The original article has been corrected.

